# Recurrent cholangiocarcinoma with long-term survival by multimodal treatment: A case report

**DOI:** 10.3892/mco.2021.2288

**Published:** 2021-04-26

**Authors:** Yuki Sota, Takahiro Einama, Kazuki Kobayashi, Ibuki Fujinuma, Takazumi Tsunenari, Yasuhiro Takihata, Toshimitsu Iwasaki, Yoichi Miyata, Koichi Okamoto, Yoshiki Kajiwara, Eiji Shinto, Hironori Tsujimoto, Shigeo Yasuda, Yuka Isozaki, Shigeru Yamada, Junji Yamamoto, Hideki Ueno, Yoji Kishi

Mol Clin Oncol 14: 72, 2021; DOI: 10.3892/mco.2021.2234

Subsequently to the publication of this article, the authors have realized that the name of the third-listed author was spelt incorrectly: The name was spelt as ‘Kazuki Kobayashibayashi’, whereas the name should have been presented as ‘Kazuki Kobayashi’. The name as it should have appeared in the author list is featured above.

The authors regret that this error was not corrected prior to the publication of the above article, and apologize to the author concerned and to the readership for any inconvenience caused.

